# Efficient hydroxylation of flavonoids by using whole-cell P450 sca-2 biocatalyst in *Escherichia coli*

**DOI:** 10.3389/fbioe.2023.1138376

**Published:** 2023-02-15

**Authors:** Baodong Hu, Xinrui Zhao, Jingwen Zhou, Jianghua Li, Jian Chen, Guocheng Du

**Affiliations:** ^1^ Key Laboratory of Industrial Biotechnology, Ministry of Education, School of Biotechnology, Jiangnan University, Wuxi, Jiangsu, China; ^2^ Science Center for Future Foods, Jiangnan University, Wuxi, Jiangsu, China; ^3^ Jiangsu Province Engineering Research Center of Food Synthetic Biotechnology, Jiangnan University, Wuxi, Jiangsu, China; ^4^ Engineering Research Center of Ministry of Education on Food Synthetic Biotechnology, Jiangnan University, Wuxi, Jiangsu, China; ^5^ Key Laboratory of Carbohydrate Chemistry and Biotechnology, Ministry of Education, Jiangnan University, Wuxi, Jiangsu, China

**Keywords:** cytochrome P450 enzyme, *Escherichia coli*, whole-cell biocatalyst, SCA-2, hydroxylation, flavonoids

## Abstract

The hydroxylation is an important way to generate the functionalized derivatives of flavonoids. However, the efficient hydroxylation of flavonoids by bacterial P450 enzymes is rarely reported. Here, a bacterial P450 sca-2_mut_ whole-cell biocatalyst with an outstanding 3′-hydroxylation activity for the efficient hydroxylation of a variety of flavonoids was first reported. The whole-cell activity of sca-2_mut_ was enhanced using a novel combination of flavodoxin Fld and flavodoxin reductase Fpr from *Escherichia coli.* In addition, the double mutant of sca-2_mut_ (R88A/S96A) exhibited an improved hydroxylation performance for flavonoids through the enzymatic engineering. Moreover, the whole-cell activity of sca-2_mut_ (R88A/S96A) was further enhanced by the optimization of whole-cell biocatalytic conditions. Finally, eriodictyol, dihydroquercetin, luteolin, and 7,3′,4′-trihydroxyisoflavone, as examples of flavanone, flavanonol, flavone, and isoflavone, were produced by whole-cell biocatalysis using naringenin, dihydrokaempferol, apigenin, and daidzein as the substrates, with the conversion yield of 77%, 66%, 32%, and 75%, respectively. The strategy used in this study provided an effective method for the further hydroxylation of other high value-added compounds.

## 1 Introduction

Cytochrome P450 enzymes (P450s, CYPs) are heme-containing enzymes that catalyze various types of chemical reactions on a variety of substrates (Hu et al., 2022b). Importantly, they are able to catalyze the regioselective and stereoselective oxidations of C-H bonds (Urlacher and Girhard, 2019). P450s are thought to be reliable, effective, and ecofriendly biocatalysts for the synthesis of valuable compounds in recombinant hosts. In addition, compared to the utilization of purified or extracted P450s, whole-cell biotransformation has shown a clear advantage by providing the necessary precursors, the expensive cofactors NAD(P)H, and suitable environments for catalytic reactions (Hu et al., 2022a). Moreover, to exploit the versatile P450s for industrial applications, *Escherichia coli* is a widely applied and efficient system for whole-cell biotransformation (Park et al., 2020).

Compared to the eukaryotic P450s, the bacterial P450s are cytosolic, presenting practical advantages for biotechnological applications (Moody and Loveridge, 2014). The largest genus of actinobacteria, *Streptomyces*, produce 70%–80% of the natural bioactive compounds (Berdy, 2005). The *Streptomyces* genomes provide a rich source of P450s that can generate a variety of novel compounds (Lamb et al., 2013). There are at least 17 subfamilies of CYP105 in *Streptomycetes*, which play important roles in the biotransformation or degradation of xenobiotics, and the biosynthesis of numerous bioactive compounds (Moody and Loveridge, 2014). For example, vitamin D_3_ can be converted to its active form (1α,25-dihydroxyvitamin D_3_) by CYP105A1 (Sawada et al., 2004). In addition, the CYP105 family has shown great potential for industrial applications (Yasuda et al., 2018). An important cholesterol-lowering drug, pravastatin, is produced by the stereoselective hydroxylation of mevastatin by CYP105A3 (P450 sca-2) in *S. carbophilus* (Watanabe et al., 1995). The activity of mutant (G52S/T85F/F89I/T119S/P159A/V194N/D269E/T323A/N363Y/E370V) increased by 29.3-fold compared to the wild type P450 sca-2 (Ba et al., 2013b). Moreover, CYP105D7 showed a broad spectrum of substrates, including pentalenic acid (Takamatsu et al., 2011), diclofenac (Xu et al., 2015), daidzein (Pandey et al., 2010), naringenin (Liu et al., 2016), compactin (Yao et al., 2017), testosterone (Ma et al., 2019), and capsaicin (Ma et al., 2021).

**FIGURE 1:**
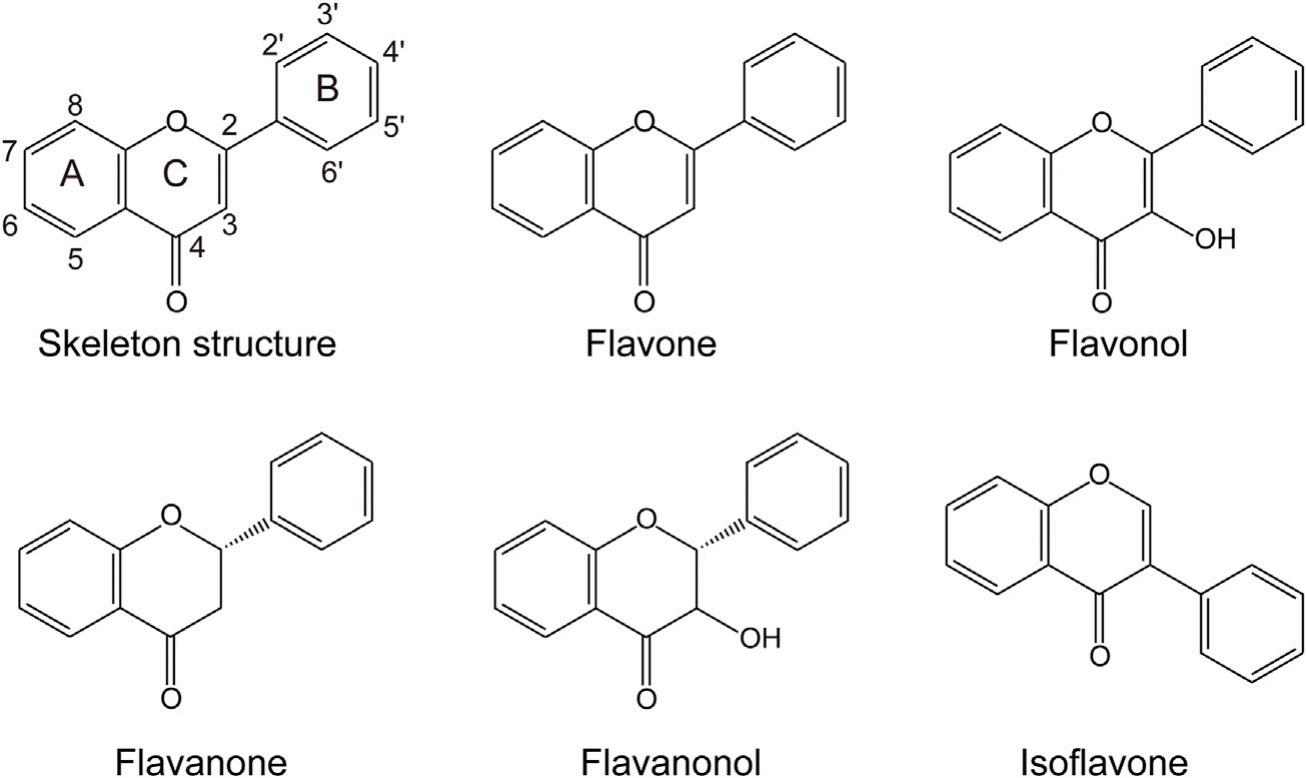
The skeleton structure of the main classes of flavonoids.

Flavonoids are one of the largest known groups of natural products, which are widely found in the plants (Havsteen, 2002). They have a phenyl benzopyrone structure (C6-C3-C6) and are mainly classified as flavones, flavanols, flavanones, flavanonols, and isoflavones (Figure 1) (Middleton et al., 2000). They exhibit therapeutic and chemo-preventive effects on human health, including antioxidant activity (PG, 2000), antimicrobial activity (Cushnie and Lamb, 2005), anti-inflammatory activity (Pan et al., 2010), and anti-cancer properties (Ravishankar et al., 2013). In addition, they can be served as potential drug candidates to treat symptoms associated with the coronavirus disease (COVID-19) infection (Adhikari et al., 2021). However, the low water solubility and instability limit the pharmaceutical application of these flavonoid compounds (Chu et al., 2016). Hydroxylation is a common strategy to improve their solubility and stability (Lin and Yan, 2014). Moreover, the structural diversity and biological activity of flavonoids also can be improved through hydroxylation. For example, 7,3′,4′-trihydroxyisoflavone, the 3′-hydroxylated product of daidzein, exhibits better anti-cancer properties than daidzein and plays an essential role in suppressing ultraviolet B-induced skin cancer (Lee et al., 2011).

Biocatalytic hydroxylation is an environmentally friendly approach compared to the chemical hydroxylation. Flavonoids 3′-hydroxylase (F3′H), responsible for the hydroxylation of flavonoids in plants, has been well studied (Gao et al., 2020; Liu et al., 2022; Park et al., 2022b). However, the plant-derived P450s have low activity in prokaryotic hosts (Gao et al., 2020; Park et al., 2022b). Currently, the hydroxylation of flavonoids has not been well achieved in bacteria. Several bacterial P450s, including P450 BM3 (Chu et al., 2016), CYP105D7 (Liu et al., 2016), CYP107P2 (Pandey et al., 2011), CYP107Y1 (Pandey et al., 2011), and CYP105A5 (Subedi et al., 2022), have been explored to hydroxylate selected flavonoids with a low conversion rate.

In this study, we report a bacterial whole-cell biocatalyst for the efficient hydroxylation of a variety of flavonoids. At first, the bacterial P450 sca-2_mut_ exhibiting outstanding 3′-hydroxylation activity towards flavonoids was selected from five P450s candidates. Then, the whole-cell activity of sca-2_mut_ towards flavonoids was enhanced by employing a new combination of redox partners and enzymatic engineering of sca-2_mut_. Subsequently, the whole-cell activity was further enhanced by the optimization of whole-cell biocatalytic conditions. Finally, the whole-cell sca-2_mut_ biocatalyst was applied to efficiently produce eriodictyol, dihydroquercetin, luteolin, 7,3′,4′-trihydroxyisoflavone as examples of flavanone, flavanonol, flavone, and isoflavone, respectively.

## 2 Materials and methods

### 2.1 Strains and reagents

*E. coli* DH5α and C41(DE3) were used as hosts for DNA cloning and whole-cell biotransformation, respectively. Primer STAR HS DNA polymerase and restriction endonucleases were obtained from Takara (Dalian, China). DNA and genomic DNA Extraction Kits were obtained from Thermo Scientific (Waltham, United States) and TIANGEN (Beijing, China), respectively. The plasmid miniprep purification kit was purchased from Sangon Biotech (Shanghai, China). Oligonucleotide synthesis and sequence analysis were achieved by Sangon Biotech (Shanghai, China). ALA and hemin were purchased from Sigma-Aldrich (St. Louis, MO, United States). Naringenin, eriodictyol, dihydrokaempferol, dihydroquercetin, kaempferol, quercetin, apigenin, luteolin, daidzein, and 7,3′,4′-trihydroxyisoflavone were purchased from Yuanye Bio-Technology (Shanghai, China). Other chemicals were purchased from Sangon Biotech (Shanghai, China) and were of the highest commercial grade available.

### 2.2 Plasmids and strains construction

All the plasmids, primers, and strains used in this study are listed in Supplementary Tables S1–S3, respectively.

The genes encoding CYP105D7 from *S. avermitilis* (Liu et al., 2016), CYP105A3 (variant III; G52S/T85F/F89I/T119S/P159A/V194N/D269E/T323A/N363Y/E370V; named sca-2_mut_) from *S. carbophilus* (Ba et al., 2013b), CYP 105P2 from *S. peucetius* (Niraula et al., 2012), CYP105A1 (R73A/R84A; named CYP105A1_mut_) from *S. griseolus* (Yasuda et al., 2017), CYP105AB3 (Q87W/T115A/H132L/R191W/G294D; named moxA_mut_) from *Nonomuraea recticatena* (Kabumoto et al., 2009), putidaredoxin reductase (CamA) and putidaredoxin (CamB) from *Pseudomonas putida* (Ba et al., 2013a) were codon optimized and synthesized by GenScript (Nanjing, China). To construct the whole-cell biocatalytic system for the hydroxylation of flavonoids, *camB-camA* genes were first subcloned into the *Nco* I*/Sal* I of plasmid pRSFDuet-1 to generate plasmid pRSF-CamA-CamB. Subsequently, *CYP105P2*, *CYP105D7*, *moxA*_*mut*_, *CYP105A1*_*mut*_, and *sca-2*_*mut*_ genes were individually subcloned into the *Nde* I*/Xho* I of plasmid pRSF-CamA-CamB to generate plasmids pRSF-105P2-CamA-CamB, pRSF-105D7-CamA-CamB, pRSF-moxA_mut_-CamA-CamB, pRSF-105A1_mut_-CamA-CamB, and pRSF-sca-2_mut_-CamA-CamB, respectively.

To investigate the effect of different redox partners on the catalytic performance of sca-2_mut_ towards flavonoids, other redox partners were selected and optimized. First, using the plasmid donated by Professor Shengying Li from Shandong University as a template, the genes encoding ferredoxin Fdx_1499 and the ferredoxin reductase FdR_0978 from *Synechococcus elongates* PCC7942 (Zhang et al., 2018) were obtained by PCR using primers Fdx_1499-RH-F/Fdx_1499-RH-R and FdR_0978-RH-F/FdR_0978-RH-R, respectively. These two obtained fragments were fused by overlap extension PCR (Horton et al., 1993), and subsequently inserted into the *Nco* I*/Sal* I of plasmid pRSF-sca-2_mut_ to generate plasmid pRSF-sca-2_mut_-Fdx_1499-FdR_0978. In the same way, the gene encoding flavodoxin reductase Fpr (GenBank: QJZ14319.1) from *E. coli* in combination with the genes encoding endogenous flavodoxin Fld (GenBank: QJZ13227.1), FldA (GenBank: QJZ11404.1) (Bakkes et al., 2015), or FldB (GenBank: QJZ13309.1), were used to construct plasmids pRSF-sca-2_mut_-Fld-Fpr, pRSF-sca-2_mut_-FldA-Fpr, and pRSF-sca-2_mut_-FldB-Fpr, respectively. Furthermore, the gene encoding *E. coli* Fpr grouped with the genes encoding flavodoxin YkuN (Gene ID: 939194) or flavodoxin YkuP (Gene ID:938811) from *Bacillus subtilis* (Bakkes et al., 2017), were inserted into the *Nco* I*/Sal* I of plasmid pRSF-sca-2_mut_ to generate plasmids pRSF-sca-2_mut_-YkuN-Fpr and pRSF-sca-2_mut_-YkuP-Fpr, respectively. In addition, the fused enzyme (sca-2_mut_-BM3) was constructed by fusing the heme domain of sca-2_mut_ and the reductase domain of P450 BM3 from *Bacillus megaterium*. Fragments of gene *sca-2*_*mut*_ and the reductase domain of *BM3* were obtained by PCR using primers Sca2-RH-F/Sca2-RH-R and BM3-RH-F/BM3-RH-R, respectively. The products of amplification were fused by overlap extension PCR and subsequently inserted into the *Nde* I*/Xho* I of pRSFDuet-1 to generate plasmid pRSF-sca-2_mut_-BM3.

To construct the mutants of sca-2_mut_ (plasmids sca-2_mut_R77A-Fld-Fpr, sca-2_mut_R88A-Fld-Fpr, sca-2_mut_R93A-Fld-Fpr, sca-2_mut_G95A-Fld-Fpr, sca-2_mut_S96A-Fld-Fpr, sca-2_mut_R197A-Fld-Fpr, and sca-2_mut_R88A/S96A-Fld-Fpr), the fragments were obtained by PCR using the primers (Supplementary Table S2) with plasmid pRSF-sca-2_mut_-Fld-Fpr as a template, and the obtained PCR products were assembled by Gibson assembly (Gibson et al., 2009).

To construct the plasmid ADB-N-sca-2_mut_R88A/S96A-Fld-Fpr, the zinc finger proteins ADB1 (RSNR-RDHT-VSTR-QSNI), ADB2 (VSSR-RSHR-RSNR-CSNR), and ADB3 (QSSR-RSHR-RHHR-QTHQ) (Xu et al., 2020) were fused to the N terminus of Fpr, Fld, and sca-2_mut_R88A/S96A, respectively (primers in Supplementary Table S2). Using the same approach, the zinc finger proteins ADB1, ADB2, and ADB3 were fused to the N terminus of Fpr, Fld, and sca-2_mut_R88A/S96A, respectively, to generate plasmid ADB-C-sca-2_mut_R88A/S96A-Fld-Fpr. To construct the plasmids Lig-N-sca-2_mut_R88A/S96A-Fld-Fpr and Lig-C-sca-2_mut_R88A/S96A-Fld-Fpr, the ligands of GBD, SH3, and PDZ were fused to the N terminus or C terminus of Fpr, Fld, and sca-2_mut_R88A/S96A, respectively (primers in Supplementary Table S2). Plasmid DNA scaffold was constructed by PCR using primers DNA-scaffold-F/DNA-scaffold-R with plasmid pACYCDuet-1 as a template. Plasmids protein scaffold, CipA-N-sca-2_mut_R88A/S96A-Fld-Fpr, CipA-C-sca-2_mut_R88A/S96A-Fld-Fpr, CipB-N-sca-2_mut_R88A/S96A-Fld-Fpr, and CipB-C-sca-2_mut_R88A/S96A-Fld-Fpr were codon optimized and synthesized by GenScript (Nanjing, China).

### 2.3 Medium and culture conditions

Luria-Bertani (LB) medium (10 g/L tryptone, 5 g/L yeast extract, 10 g/L NaCl, and pH 7.0) was used for cloning and seeding cultures. To obtain seed cultures, colonies of the recombination strain grown from LB agar plates (2% agar, w/v) were inoculated into 50 mL test tubes containing 5 mL LB medium supplemented with 50 μg/mL kanamycin and incubated in a rotary shaker at 37°C and 220 rpm for 12 h. 1 mL of the seed cultures was transferred to 250 mL shaking flasks containing 50 mL Terrific Broth (TB) medium (12 g/L tryptone, 24 g/L yeast extract, 0.4% v/v glycerol, 0.017 M KH_2_PO_4_, and 0.072 M K_2_HPO_4_) supplemented with 50 μg/mL kanamycin, 100 mg/L ALA and 20 mg/L FeSO_4_·7H_2_O, and the cultures were then incubated at 37°C and 220 rpm. When the optical density at 600 nm (OD_600_) reached 0.6-0.8, 1 mM isopropyl β-D-1-thiogalactopyranoside (IPTG) was added to induce enzyme expression. After induction, the cultures were incubated at 25°C for 20 h.

For cultivation of HFLA-20 to HFLA-23 strains, 50 μg/mL kanamycin and 34 μg/mL chloramphenicol were added into the medium.

### 2.4 The hydroxylation of flavonoids by whole-cell biocatalysis

After cultivation, 50 mL of cells were harvested by centrifugation (8,000 rpm, 10 min), then washed twice with potassium phosphate buffer (100 mM, pH 8.0), and subsequently resuspended with 25 mL potassium phosphate buffer (100 mM, pH 8.0) containing 10% glycerol or 10% glucose. 25 mL of cell suspension (30 OD_600_) was used for the whole-cell biocatalysis in 250 mL shaking flasks.

To examine the catalytic efficiency of hydroxylation of flavonoids, naringenin, dihydrokaempferol, kaempferol, apigenin, and daidzein (5 g/L in ethanol) was added to the cell suspension to give the final concentration of 100 mg/L, respectively. Whole-cell biocatalysis were performed at 30°C and 220 rpm for 12 h. Then, 1 mL of the whole-cell biocatalytic reaction solution was collected and extracted thrice with 1 mL ethyl acetate. The products were dried, dissolved in methanol, and subsequently analyzed using high-performance liquid chromatography (HPLC).

### 2.5 The optimal conditions for the hydroxylation of flavonoids by whole-cell biocatalysis

To optimize biocatalytic conditions, 25 mL of the cell suspension (30 of OD_600_) was used for the bioconversion reaction in 250 mL shaking flasks. To investigate the effect of temperature on the catalytic activity, reactions were performed at pH 8.0 with the temperature ranging from 20°C to 40°C. To optimize pH, reactions were performed at 37°C and 220 rpm in potassium phosphate buffer (pH 6.0-8.0) or Tris-HCl buffer (pH 9.0).

### 2.6 Homology modelling and ligand docking

A homology model of sca-2_mut_ and sca-2_mut_R88A/S96A was constructed using the highly homologous template CYP105A1 (PDB code 2ZBX, 75.1% identity) (Sugimoto et al., 2008) in Discovery Studio 2019 (DS 2019). The predicted structures for sca-2_mut_ and sca-2_mut_R88A/S96A were evaluated by UCLA−DOE LAB-SAVES v6.0 web server (https://saves.mbi.ucla.edu/). Molecular docking analysis was performed using the CDOCKER tool of DS 2019.

### 2.7 Analytical procedures

Cell growth was detected by measuring OD_600_ using a spectrophotometer (UVmini-1240, Shimadzu Corporation, Japan).

Naringenin, eriodictyol, dihydrokaempferol, dihydroquercetin, kaempferol, quercetin, apigenin, luteolin, daidzein, and 7,3′,4′-trihydroxyisoflavone were quantified using an Agilent 1260 HPLC instrument (Agilent Technologies, Santa Clara, CA, United States) equipped with ultraviolet/VIS detector. A reverse-phase column ZORBAX Eclipse XDB-C18 (5 μm, 4.6 mm × 250 mm, Agilent, United States) was used to monitor the absorbance at 290 nm. Elution was performed with mobile phase A consisting water containing 0.1% trifluoroacetic acid and mobile phase B consisting methanol containing 0.1% trifluoroacetic acid. The flow rate was set as 0.8 ml·min^-1^ and the solvent gradient was adopted as follow: 0–1 min, isocratic at 10% B; 1–10 min, 10%–40% B; 10–20 min, 40%–60% B; 20–23 min, 60% B; 23–25 min, 60%–10% B; 25–27 min, 10% B.

### 2.8 Data analysis

All experiments were performed with three biological replicates. The data were analyzed by the software GraphPad Prism 8.0 and displayed as mean values ± standard deviation (SD) from triplicate experiments.

## 3 Results and discussion

### 3.1 Screening of the efficient bacterial P450s for the hydroxylation of flavonoids

**FIGURE 2:**
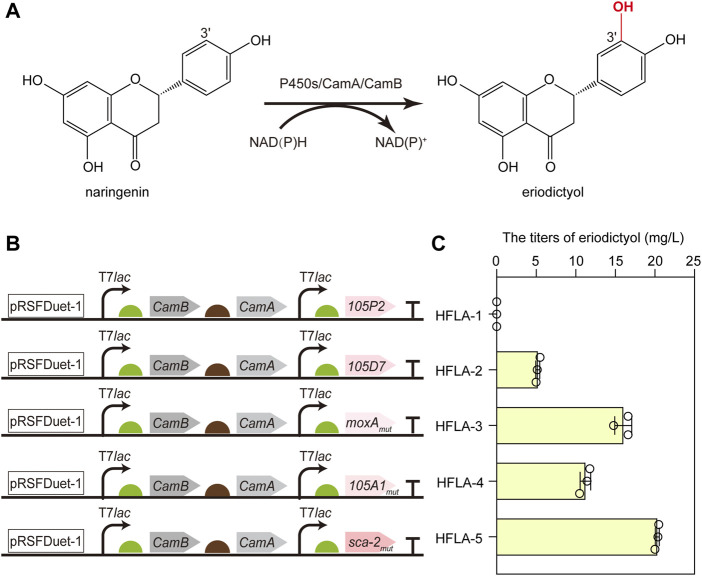
Screening of the efficient bacterial P450s for the hydroxylation of flavonoids. **(A)** Schematic representation of the regioselective hydroxylation of naringenin by P450s with redox partners of CamA and CamB. **(B)** Schematic diagram of combining different P450s and redox partners to construct whole-cell biocatalyst plasmids. P450s included CYP105P2, CYP105D7, P450 moxA_mut_, CYP105A1_mut_, and P450 sca-2_mut_. **(C)** Titers of eriodictyol produced by whole-cell biocatalysts of different P450s using 100 mg/L naringenin. The data are shown as mean ± SD of three biological replicates.

To screen the efficient bacterial P450s for the hydroxylation of flavonoids, CYP105D7 from *S. avermitilis* (Liu et al., 2016), sca-2_mut_ (CYP105A3) from *S. carbophilus* (Ba et al., 2013b), CYP105P2 from *S. peucetius* (Niraula et al., 2012), CYP105A1_mut_ from *S. griseolus* (Yasuda et al., 2017), and moxA_mut_ (CYP105AB1) from *N. recticatena* (Kabumoto et al., 2009) were chosen as candidates to investigate the catalytic performance toward flavonoids. Since the CYP105 family is a three-component P450 enzyme, the most widely studied redox partners CamA (putidaredoxin reductase) and CamB (putidaredoxin) were employed in the whole-cell biocatalysis to transfer electrons from NAD(P)H to the heme-iron reactive center for O_2_ activation. Thus, the genes encoding CamA and CamB were co-expressed with these five P450s genes using a pRSFDuet-1 plasmid in the C41(DE3) strain (Hu et al., 2022a), respectively, resulting in HFLA-1 to HFLA-5 strains. Subsequently, the hydroxylation of flavonoids by whole-cell as biocatalysts was compared using naringenin as a model substrate (Figures 2A, B).

Except the HFLA-1 strain (harboring plasmid pRSF-105P2-CamA-CamB), the other four strains can catalyze hydroxylation at C-3′ of naringenin to produce eriodictyol (Figure 2C). A titer of 5.2 ± 0.3 mg/L eriodictyol was produced by the HFLA-2 strain (harboring plasmid pRSF-105D7-CamA-CamB) using 100 mg/L of naringenin as a substrate, which was comparable to that previously reported (10.3% of conversion rate using 0.15 mM naringenin) (Liu et al., 2016). Since the quintuple mutant Q87W/T115A/H1432L/R194W/G294D showed 4.3-fold higher activity towards naringenin than the wild-type moxA (Kabumoto et al., 2009), a higher level of eriodictyol (16.0 ± 1.1 mg/L) was obtained by the HFLA-3 strain (harboring plasmid pRSF-moxA_mut_-CamA-CamB). Furthermore, 11.2 ± 0.7 and 20.3 ± 0.3 mg/L eriodictyol were produced by the HFLA-4 strain (harboring plasmid pRSF-105A1_mut_-CamA-CamB) and the HFLA-5 strain (harboring plasmid pRSF-sca-2_mut_-CamA-CamB), respectively, which were the first report of eriodictyol production by CYP105A1 and P450 sca-2 through hydroxylation at the C-3′ of naringenin. In particular, sca-2_mut_ showed the best catalytic performance in the C-3′ hydroxylation of naringenin, and the titer of eriodictyol produced by the HFLA-5 strain was 3.9-, 1.3-, and 1.8-fold higher than that produced by the HFLA-2 strain, the HFLA-3 strain, and the HFLA-4 strain. Therefore, sca-2_mut_ was selected for efficient hydroxylation of flavonoids.

### 3.2 Improving the catalytic activity of sca-2_mut_ by engineering redox partners

**FIGURE 3:**
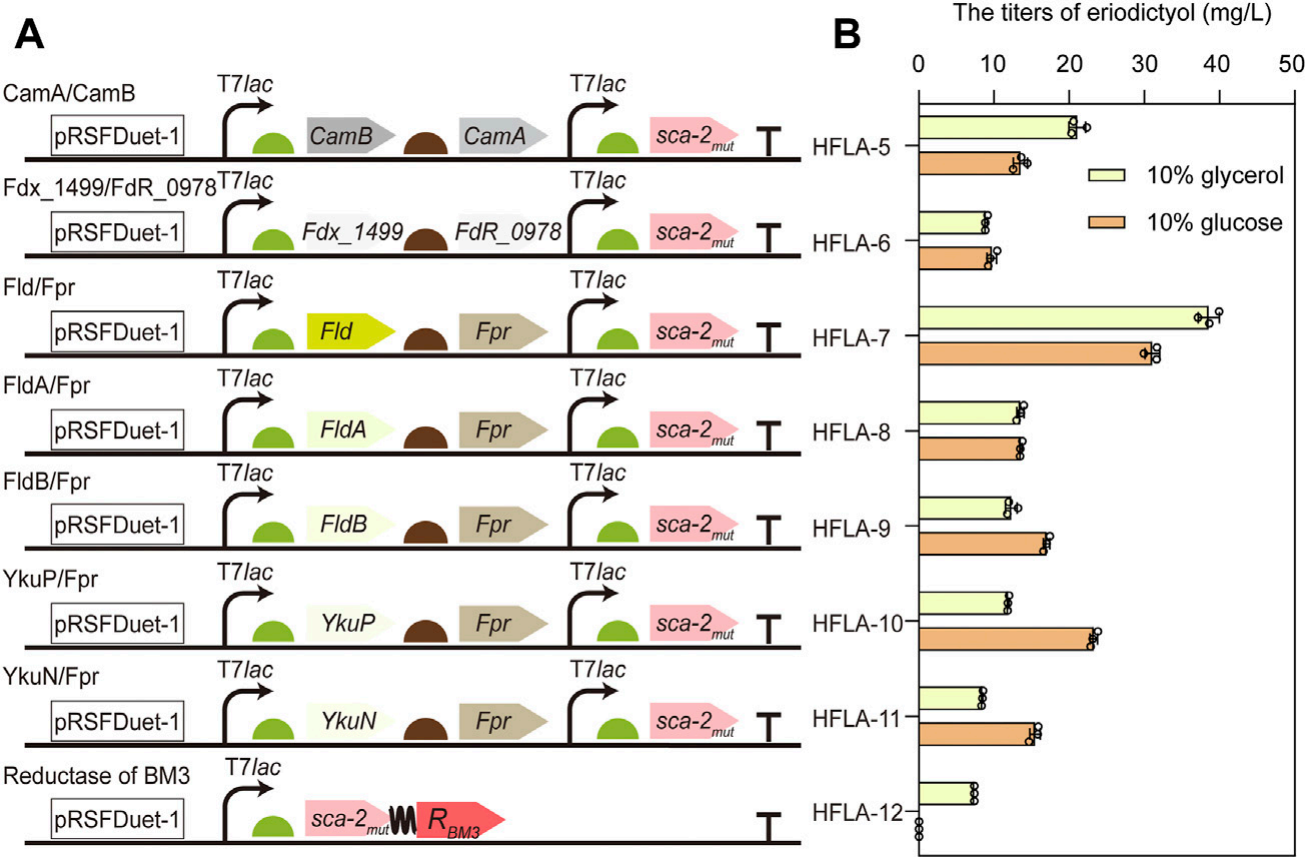
Improving the catalytic activity of sca-2_mut_ by engineering redox partners. **(A)** Schematic representation of combining sca-2_mut_ with different redox partners to reconstitute the activity of sca-2_mut_. Redox partners included Fdx_1499/FdR_0978, Fld/Fpr, FldA/Fpr, FldB/Fpr, YkuP/Fpr, YkuN/Fpr, and the reductase domain of BM3. **(B)** Titers of eriodictyol produced by different sca-2_mut_ whole-cell biocatalysts with different redox partners using 100 mg/L naringenin. The data are shown as mean ± SD of three biological replicates.

For the common bacterial three-component P450s, the process of transferring electron by redox partner is important for catalysis. However, the optimal redox partners for sca-2_mut_ are unknown (Zhang et al., 2018). To obtain the suitable redox partners for the hydroxylation of flavonoids by P450 sca-2_mut_, the different combinations of flavodoxin and flavodoxin reductase were used to reconstitute the activity of P450 sca-2_mut_, including the electron transfer proteins flavodoxin (Fld, FldA, or FldB) from *E. coli* in combination with the endogenous flavodoxin reductase Fpr (Bakkes et al., 2015), respectively, the flavodoxin (YkuN or YkuP) (Girhard et al., 2010) from *B. subtilis* in combination with *E. coli* Fpr, respectively, and the ferredoxin Fdx_1499 and ferredoxin reductase FdR_0978 from *S. elongates* PCC7942 (Sun et al., 2017) (Figure 3A). In addition, the chimeric protein was constructed by fusing the P450 sca-2_mut_ to the reductase domain of P450BM3 from *B. megaterium* (Figure 3A).

The plasmids harboring the genes encoding different redox partners were transformed into the C41(DE3) strain, resulting in HFLA-6 to HFLA-12 strains. Subsequently, their performances in the hydroxylation of flavonoids were investigated using a final concentration of 100 mg/L naringenin as a substrate. Whole-cell biocatalysis was performed in potassium phosphate buffer containing glucose (1%, 2%, and 10% w/v) or glycerol (10% v/v), respectively. The seven strains showed similar catalytic activity toward naringenin in the biocatalytic systems containing 1%, 2%, and 10% w/v of glucose (Supplementary Figure S1). The HFLA-7 strain (harboring plasmid pRSF-sca-2_mut_-Fld-Fpr) had both the best catalytic performance towards naringenin in glycerol or glucose containing biocatalytic system, producing 38.6 ± 1.4 or 31.1 ± 1.0 mg/L eriodictyol, respectively (Figure 3B). This result suggested that glycerol was more beneficial to the catalytic efficiency than glucose in the whole-cell biocatalysis of HFLA-7 strain. Notably, the *E. coli* flavodoxin Fld was used in combination with Fpr for the first time to reconstitute the activity of P450s. In the recent study, FdR_0978/Fdx_1499 was the most promising redox partner for the *in vitro* activity of sca-2 towards mevastatin compared to the redox systems Adx/AdR and Pdx/PdR (Liu et al., 2022). However, the HFLA-6 strain (harboring plasmid pRSF-sca-2_mut_-Fdx_1499-FdR_0978) produced 8.9 ± 0.2 and 9.7 ± 0.6 mg/L eriodictyol in the *in vivo* biocatalytic system containing glycerol or glucose, which were only 23.1% and 31.2% of the HFLA-7 strain, respectively (Figure 3B). Therefore, the HFLA-7 strain was used for the following improvement.

### 3.3 Enhancing the catalytic activity of sca-2_mut_ by sequence-guided engineering

**FIGURE 4:**
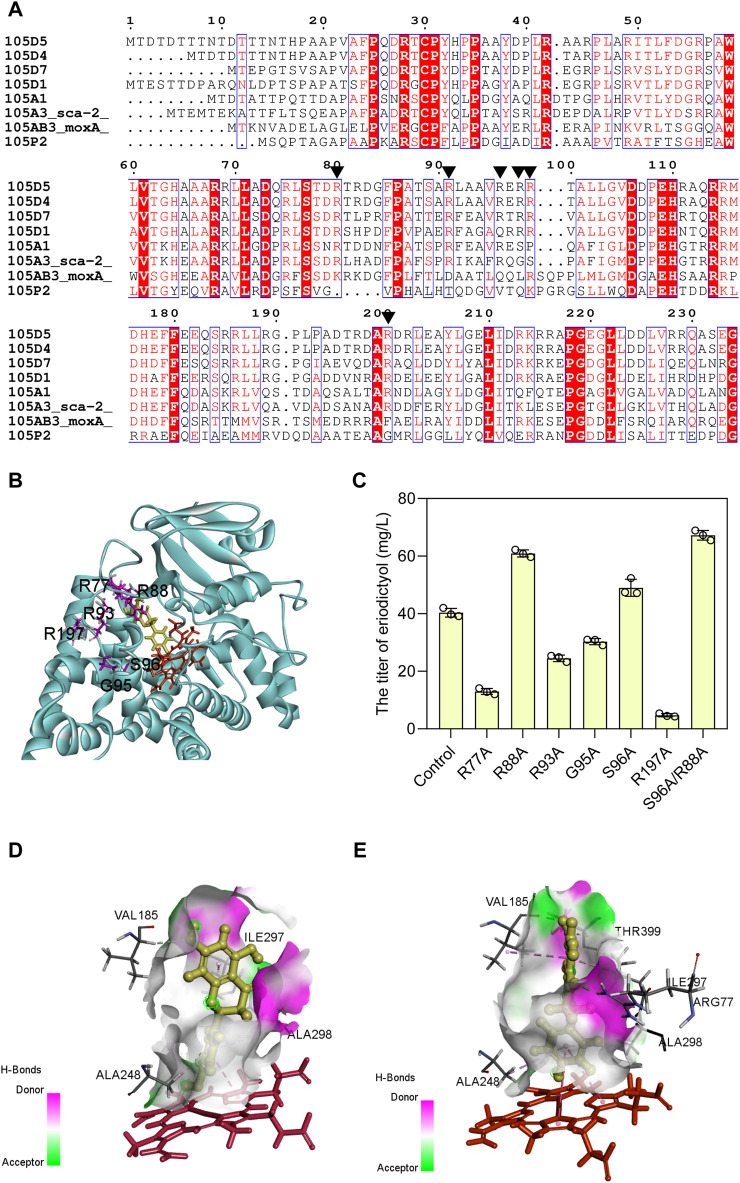
Enhancing the catalytic activity of sca-2_mut_ by sequence-guided engineering. **(A)** Amino acid sequence alignment using the representative CYP105 family. Black triangle indicates the conserved arginine residues on the surface of an active site. **(B)** Selection of amino acids used as mutations (R77, R88, R93, G95, S96, and R197) are positioned around the substrate area of sca-2. **(C)** Titers of eriodictyol produced by engineering strains containing sca-2 mutants. **(D)** The interactions between naringenin and sca-2_mut_ model. **(E)** The interactions between naringenin and sca-2_mut_R88A/S96A model. Heme was indicated as red and ligand indicated as yellow. The data are shown as mean ± SD of three biological replicates.

Based on the evolutionary information encapsulated in homologous protein sequences, the approach of consensus design has been employed to improve the stability (Porebski and Buckle, 2016) and activity (Yao et al., 2022) of proteins. Hence, the mutagenesis was designed to obtain the sca-2 variants by consensus design. Multiple sequence alignment was performed on the CYP105 family and the conserved arginine residues around the active site pocket were shown as red boxes (Figure 4A). CYP105D7, sharing 53% identity of amino acid sequence with sca-2, has four arginine residues (Arg70, Arg81, Arg88, and Arg190) that form a wall of the substrate-binding pocket (Xu et al., 2015), and the double mutant R70A/R190A has a nearly 9-fold increase in the *in vivo* conversion rate of testosterone (Ma et al., 2019). The distal pocket of CYP105A1 contains three Arg residues (Arg73, Arg84, and Arg193) (Sugimoto et al., 2008), and the double mutant R73A/R84A exhibited a 319-fold higher *K*_*cat*_ */K*_*m*_ for 25-hydroxylation towards the substrate 1α(OH) vitamin D_3_ (Hayashi et al., 2008). Based on these simulated results, six residues of sca-2_mut_ (Arg77, Arg88, Arg93, Gly95, Ser 96, and Arg197) (Figure 4B) were mutated, and the catalytic performance of the mutants on naringenin were examined.

Two single mutants, sca-2_mut_R88A (HFLA-14 strain) and sca-2_mut_S96A (HFLA-17 strain), exhibited 58% and 27% significantly increase in catalytic activity for naringenin compared to the HFLA-7 strain (harboring plasmid pRSF-sca-2_mut_-Fld-Fpr), producing 60.9 ± 1.2 and 49.0 ± 2.9 mg/L eriodictyol using 100 mg/L naringenin as a substrate, respectively (Figure 4C). Then, the double mutant sca-2_mut_R88A/S96A (HFLA-19 strain) was constructed, and the catalytic activity was further increased by 10% compared to the best single mutant sca-2_mut_R88A (HFLA-14 strain), producing 67.2 ± 1.7 mg/L eriodictyol.

To analyze the reason for the enhanced yield, the homology models for the sca-2_mut_ and mutant sca-2_mut_R88A/S96A were constructed and checked (Supplementary Figure S2). Subsequently, the analysis of molecular docking was performed using the CDOCKER tool of DS 2019 with substrate naringenin as the ligand (Figures 4D, E). Although the amino acids Arg 88 and Ser 96 did not directly interact with the substrate naringenin, the hydrogen bond around the substrate increased in the sca-2_mut_ R88A/S96A model compared to the sca-2_mut_ model (Figures 4D, E; Supplementary Figure S3). In addition, the increased hydrophobic interaction of Ala 88 and Ala 96 with surrounding amino acids may lead to a more flexible of substrate access (Supplementary Figure S4).

### 3.4 Improving the efficiency of electron transfer by introducing different scaffolds

**FIGURE 5:**
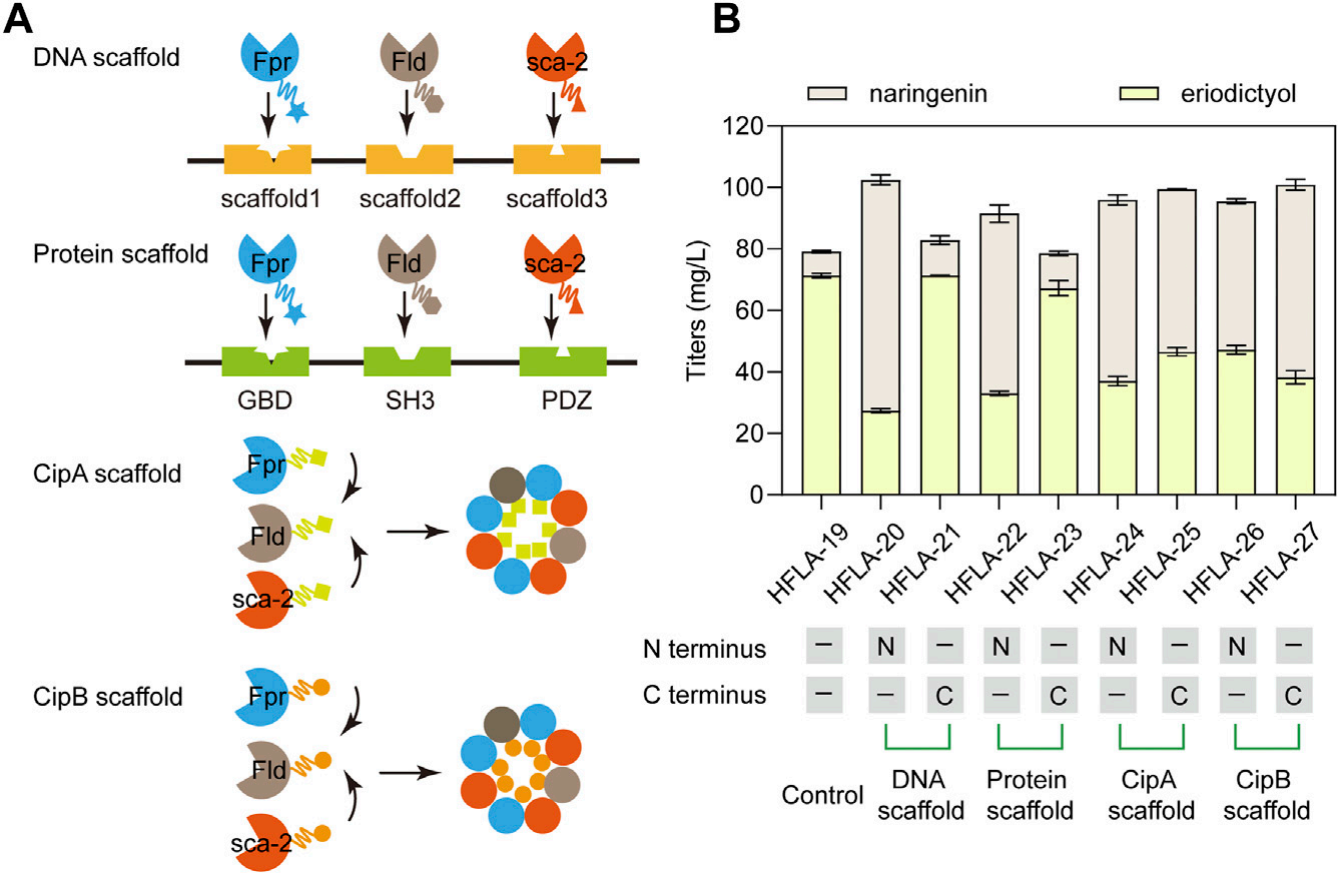
Improvement of the electron transfer efficiency by different scaffolds. **(A)** Schematic representation of the assembly of P450s and redox partners by the DNA scaffolds, Protein scaffolds, CipA scaffold, and CipB scaffold, respectively. **(B)** Titers of eriodictyol produced by different sca-2_mut_ whole-cell biocatalysts with the assembly of enzymes by scaffolds using 100 mg/L naringenin. The data are shown as mean ± SD of three biological replicates.

The efficient electron transfer between P450s and redox partners is important for the biosynthesis of natural products (Park et al., 2022a). Therefore, DNA scaffolds (Xu et al., 2020), Protein scaffolds (Dueber et al., 2009), *Photorhabdus luminescens* CipA scaffold (Wang et al., 2017), and *P. luminescens* CipB scaffold (Wang et al., 2017) were applied to assemble Fpr, Fld, and sca-2_mut_R88A/S96A. Different scaffolds were fused to the N terminus or C terminus of these three enzymes and their effects on the catalytic performance of naringenin were investigated. At first, DNA scaffolds, protein scaffolds, CipA scaffold, and CipB scaffold were respectively fused to the N terminus of Fpr, Fld, and sca-2_mut_R88A/S96A in the HFLA-19 strain to generate HFLA-20, HFLA-22, HFLA-24, and HFLA-26 strains (Figure 5A). The titers of eriodictyol in these four strains decreased by 33.7%–61.7% compared to the HFLA-19 strain, indicating that the scaffolds fused to the N terminus of the three enzymes resulted in an overall decrease in whole-cell activity (Figure 5B).

Then, the scaffolds were fused to the C terminus of Fpr, Fld, and sca-2_mut_R88A/S96A in the HFLA-19 strain, respectively, obtaining the HFLA-21, HFLA-23, HFLA-25, and HFLA-27 strains. These four strains produced 100.1%, 94.8%, 65.3%, and 53.7% of the eriodictyol titers of the HFLA-19 strain, respectively, indicating that the use of scaffolds to assemble of P450s and redox partners did not further improve the titers of eriodictyol (Figure 5B). In the previous reports, these scaffolds were used to assemble enzymes to enhance the synthesis of natural products in growing cells (Dueber et al., 2009; Xu et al., 2020; Park et al., 2022a). During the fermentation of growing cells, products are synthesized from growth substrates by the natural metabolism of the host cells and are accompanied in the fermentation broth by metabolic intermediates that make downstream processing complicated (Ladkau et al., 2014; Lee and Kim, 2015). In biotransformation, the cell growth and production phase are separated, and the use of resting cells can convert substrates to desired products (Lin and Tao, 2017). In addition, the use of resting cells is a good alternative when the optimal pH, temperature, or medium composition for biotransformation differs from the values that allow optimal growth conditions (de Carvalho, 2017). Thus, the scaffolds were not suitable for the whole-cell catalysis with P450 sca-2_mut_R88A/S96A using resting cells.

### 3.5 The optimization of whole-cell biocatalytic conditions for the HFLA-19 strain

**FIGURE 6:**
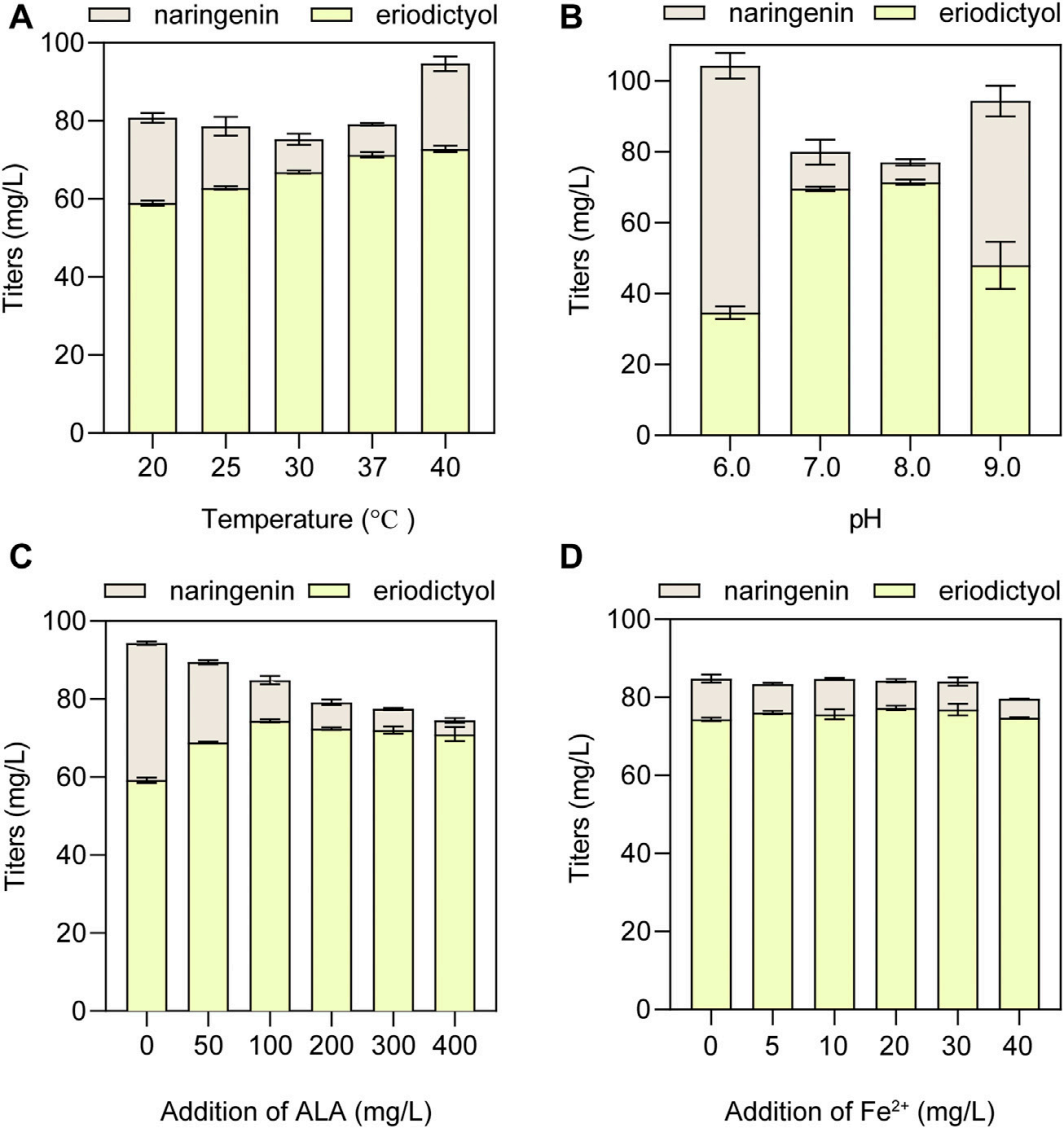
The optimization of whole-cell biocatalytic conditions for the HFLA-19 strain. **(A)** Effect of temperature on eriodictyol titer. **(B)** Effect of pH on eriodictyol titer. **(C)** Effect of ALA addition. **(D)** Effect of ferrous ion addition. The data are shown as mean ± SD of three biological replicates.

To obtain the optimal conditions of whole-cell biocatalysis, the suitable temperature and pH were optimized using the HFLA-19 biocatalyst. At first, the effect of biocatalytic temperature ranging from 20°C to 40°C on the production of eriodictyol was examined using 100 mg/L naringenin as a substrate. The results showed that the titer of eriodictyol increased with the increase of biocatalytic temperature (Figure 6A). 71.3 ± 0.7 mg/L of eriodictyol was produced at the biocatalytic temperature at 37°C, and the titer did not increase when the biocatalytic temperature was higher than 37°C. Subsequently, the effect of pH values ranging from 6.0 to 9.0 on the production of eriodictyol was examined, and the highest titer of eriodictyol (71.3 ± 0.7 mg/L) was obtained at pH 8.0°C and 37°C (Figure 6B).

Since P450s are heme-containing enzymes, increasing the intracellular supply of heme can enhance the overall activity of whole-cell biocatalysts (Park and Choi, 2020). However, the direct addition of different final concentrations of heme (5, 10, 20, 30, and 40 mg/L) to the medium did not significantly increase the titer of eriodictyol due to the weak import of heme in the C41(DE3) strain (Supplementary Figure S5), which is consistent with the previous study (Zhao et al., 2022). Since the uptake of the heme precursor (5-aminolevulinic acid, ALA) was efficient in *E. coli* (Verkamp et al., 1993), the effect of adding different final concentrations of ALA (50, 100, 200, 300, and 400 mg/L) on biocatalysis was investigated. The highest titer of eriodictyol could reach 74.3 ± 0.4 mg/L when 100 mg/L ALA was added to the medium (Figure 6C). In addition, the supplementation with iron also helps in the synthesis of heme. Based on adding 100 mg/L ALA in the medium, different final concentrations of FeSO_4_·7H_2_O (5, 10, 20, 30, and 40 mg/L) were added into the medium, respectively, and 77.3 ± 0.6 mg/L eriodictyol was produced when 20 mg/L FeSO_4_·7H_2_O was supplied (Figure 6D). Hence, the 72.5% of molar conversion rate is higher than the highest molar conversion rate (59.3%) reported so far for the heterologous expression of the F3′H from *Gentiana triflora* and cytochrome P450 reductase from *Arabidopsis thaliana* in engineering *E. coli* (Table 1) (Liu et al., 2022).

**TABLE 1:** The overview of known recombinant *Escherichia coli* whole-cell P450 biocatalysts used for the production of flavonoids.

Enzymes	Sources	Substrates (mg/L)	Titers (mg/L)	Reaction time (h)	Yields (g/g)	References
Conversion of naringenin to eriodictyol						
SPtrTT7	*A. thaliana*	100	19.7	48	0.20	Park et al. (2022b)
tr F3′H	*G. triflora*	100	62.8	10	0.63	Liu et al. (2022a)
CYP105D7	*S. avermitilis*	40.8	4.2	6	0.10	Liu et al. (2016)
CYP105D7	*S. avermitilis*	100	5.2	12	0.05	This study
P450 BM3_mut_	*B. megaterium*	27.2	11.89	48	0.44	Chu et al. (2016)
sca-2_mut_R88A/S96A	*S. carbophilus*	100	77.26	12	0.77	This study
Conversion of dihydrokaempferol to dihydroquercetin						
sca-2_mut_R88A/S96A	*S. carbophilus*	100	66.26	12	0.66	This study
Conversion of kaempferol to quercetin						
sca-2_mut_R88A/S96A	*S. carbophilus*	100	5.65	12	0.06	This study
Conversion of apigenin to luteolin						
CYP107P2	*S. avermitilis*	27	0.81	72	0.03	Pandey et al. (2011)
sca-2_mut_R88A/S96A	*S. carbophilus*	100	31.77	12	0.32	This study
Conversion of daidzein to 7,3′,4′-trihydroxyisoflavone						
CYP105D7	*S. avermitilis*	127	1.27	6	0.01	Pandey et al. (2010)
CYP105D7	*S. avermitilis*	100	27.8	12	0.28	Hu et al. (2022a)
Artificial CYP105D7	*S. avermitilis*	25.4	0.53	24	0.02	Choi et al. (2012)
sca-2_mut_R88A/S96A	*S. carbophilus*	100	75.1	12	0.75	This study

### 3.6 Sca-2_mut_R88A/S96A shows efficient catalytic performance towards other flavonoids

**FIGURE 7:**
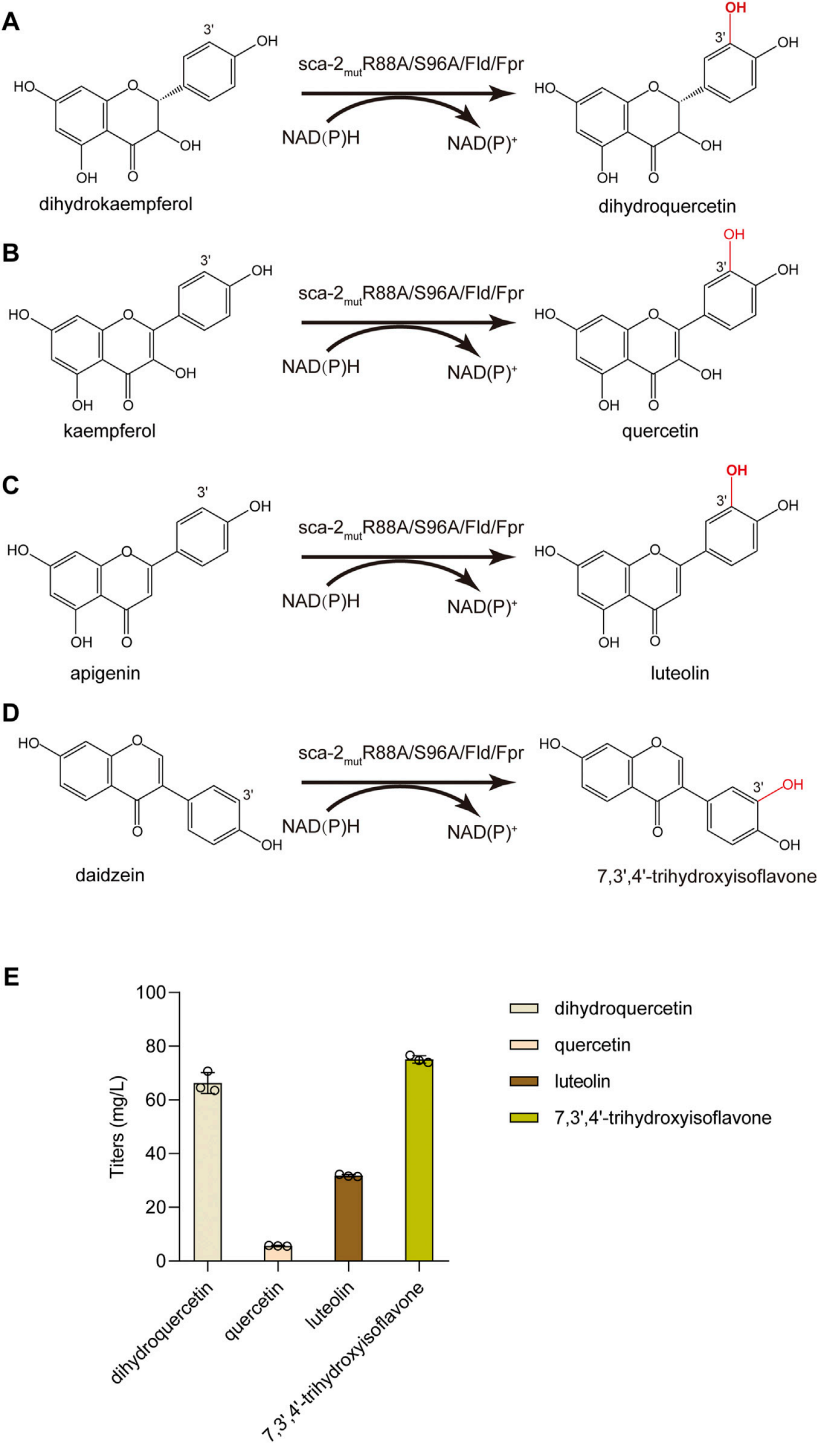
Biocatalytic hydroxylation of different flavonoids. **(A–D)** Schematic representation of the enzymatic reactions catalyzed by sca-2_mut_R88A/S96A. **(E)** Titers of dihydroquercetin, quercetin, luteolin, and 7,3′,4′-trihydroxyisoflavone produced by the HFLA-19 whole-cell biocatalyst. The data are shown as mean ± SD of three biological replicates.

Besides naringenin (flavanone), the hydroxylation potential of sca-2_mut_R88A/S96A toward dihydrokaempferol (flavanonol), kaempferol (flavonol), apigenin (flavone), and daidzein (isoflavone) was investigated. The results of whole-cell biocatalysis of the HFLA-19 strain toward the four types of flavonoids were shown in Figure 7. The HFLA-19 whole-cell biocatalyst produced 66.3 ± 3.9 mg/L of dihydroquercetin (yield: 0.66 g/g), 5.7 ± 0.1 mg/L of quercetin (yield: 0.06 g/g), 31.8 ± 0.4 mg/L of luteolin (yield: 0.32 g/g), and 75.1 ± 1.4 mg/L of 7,3′,4′-trihydroxyisoflavone (yield: 0.75 g/g), respectively, using 100 mg/L of dihydrokaempferol, kaempferol, apigenin, and daidzein as the substrates. The HFLA-19 whole-cell biocatalyst showed the efficient catalytic performance of C-3′ hydroxylation toward dihydrokaempferol, apigenin, and daidzein compared to the previously reported (Table 1). Notably, the conversion rates of dihydrokaempferol and daidzein by the HFLA-19 whole-cell catalysis were the highest reported so far.

## 4 Conclusion

In this study, an efficient bacterial whole-cell P450 biocatalyst was obtained by mining the suitable P450 enzymes, engineering redox partners, protein engineering, and the optimization of whole-cell biocatalytic conditions. By using the sca-2_mut_R88A/S96A whole-cell biocatalyst, eriodictyol, dihydroquercetin, quercetin, luteolin, and 7,3′,4′-trihydroxyisoflavone were produced with the titers of 77.3, 66.3, 5.7, 31.8, and 75.1 mg/L, respectively, in a reaction system containing a final concentration of 100 mg/L substrate. To the best of our knowledge, this is the first report of C-3′ hydroxylation of flavonoids by P450 sca-2 (CYP105A3), expanding the substrate spectrum of sca-2. In addition, the conversion rates of eriodictyol, dihydroquercetin, luteolin, and 7,3′,4′-trihydroxyisoflavone were the highest conversion rates obtained so far by whole-cell biocatalysis of P450s. This study demonstrates a versatile P450 whole-cell biocatalyst for the efficient hydroxylation of flavonoids, providing a potential biocatalyst for application in synthetic biology.

## Data Availability

The original contributions presented in the study are included in the article/Supplementary Material, further inquiries can be directed to the corresponding authors.
