# Supplementary material for: Efficient hydroxylation of flavonoids by using whole-cell P450 sca-2 biocatalyst in Escherichia coli
Source: Front Bioeng Biotechnol. 2023 Feb 15;11:1138376. doi: 10.3389/fbioe.2023.1138376 (PMC9977193; doi:10.3389/fbioe.2023.1138376)
Supplement: Supplementary file 1 [file DataSheet1.docx]

**Supplementary Information**

**Efficient hydroxylation of flavonoids by using whole-cell P450 sca-2 biocatalyst in *Escherichia coli***

**Baodong Hu ^1,2,3,4^, Xinrui Zhao ^1,2,3,4*^, Jingwen Zhou ^1,2,3,4^, Jianghua Li ^1,2,3,4^, Jian Chen ^1,2,3,4^, Guocheng Du ^1,2,3^****^,4,5*^**

^1^ Key Laboratory of Industrial Biotechnology, Ministry of Education, School of Biotechnology, Jiangnan University, 1800 Lihu Road, Wuxi, Jiangsu 214122, China;

^2^ Science Center for Future Foods, Jiangnan University, 1800 Lihu Road, Wuxi, Jiangsu 214122, China;

^3^ Jiangsu Province Engineering Research Center of Food Synthetic Biotechnology, Jiangnan University, 1800 Lihu Road, Wuxi, Jiangsu 214122, China;

^4^ Engineering Research Center of Ministry of Education on Food Synthetic Biotechnology, Jiangnan University, 1800 Lihu Road, Wuxi, Jiangsu 214122, China;

^5^ Key Laboratory of Carbohydrate Chemistry and Biotechnology, Ministry of Education, Jiangnan University, 1800 Lihu Road, Wuxi, Jiangsu 214122, China

**^*^ Correspondence:** zhaoxinrui@jiangnan.edu.cn; gcdu@jiangnan.edu.cn

Mailing address: School of Biotechnology, Jiangnan University, 1800 Lihu Road, Wuxi, Jiangsu 214122, China.

**Supplementary Figures**

**
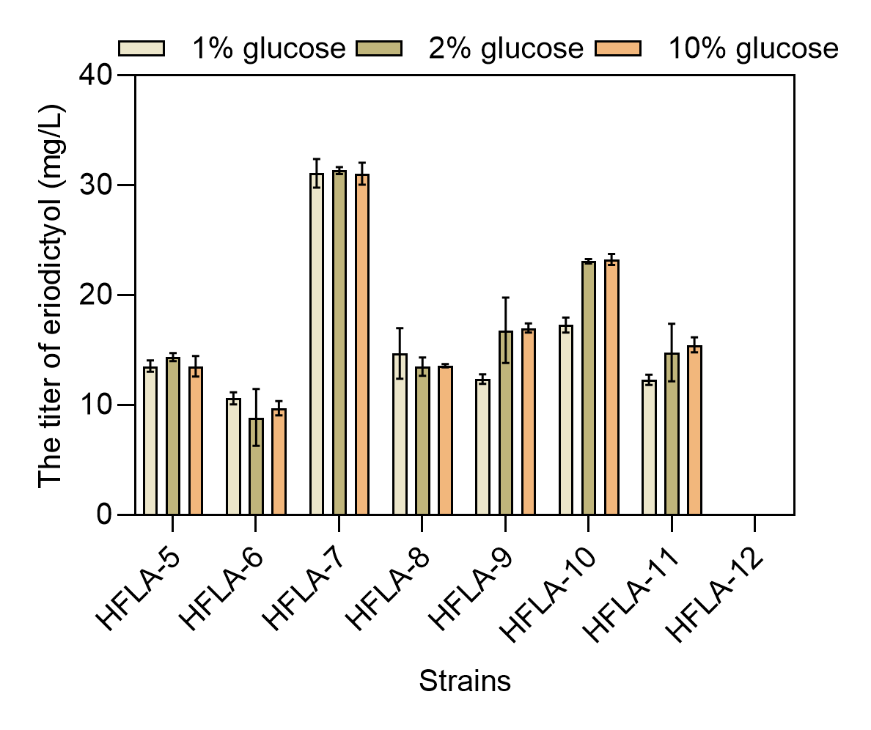
**

**Figure S1.** Catalytic performance of sca-2_mut_ with different redox partners in various biocatalytic systems containing different concentrations of glucose. The data are shown as mean ± SD of three biological replicates.

**
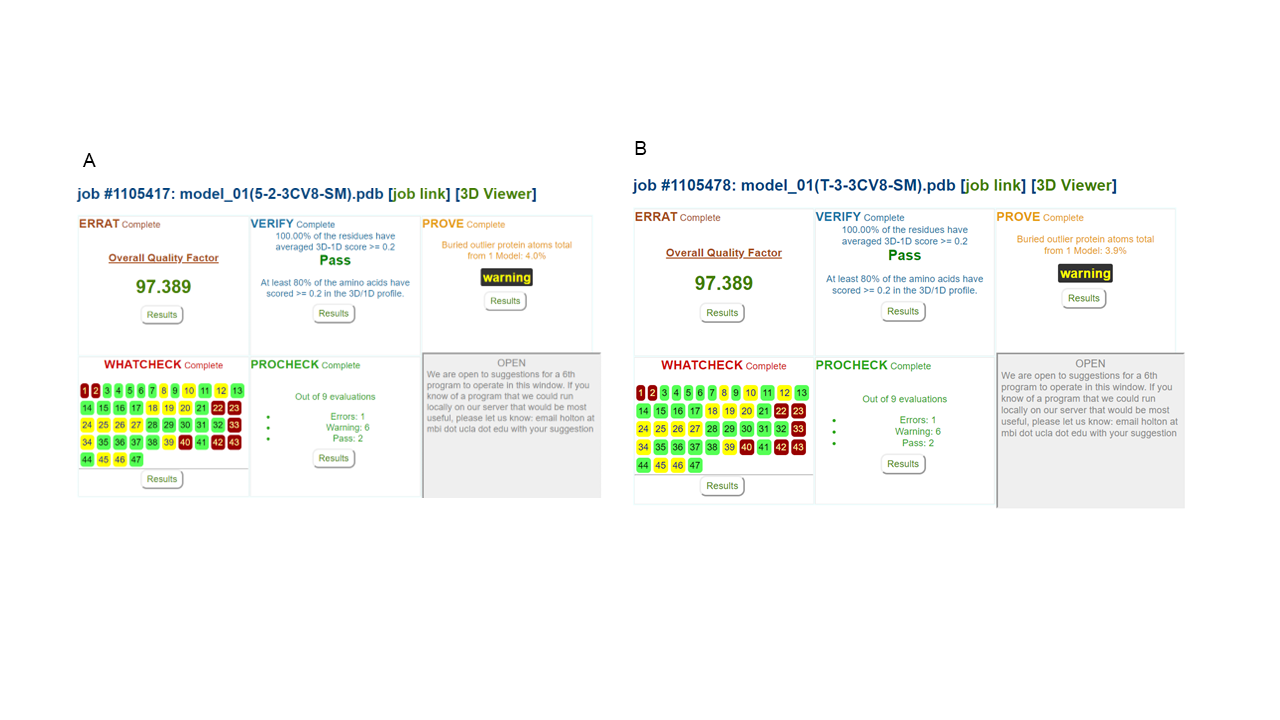
**

**Figure S2.** The evaluation of predicted models by UCLA−DOE LAB-SAVES v6.0 web server. (**A**) the predicted sca-2_mut_ model. (**B**) the predicted sca-2_mut_R88A/S96A model.

**
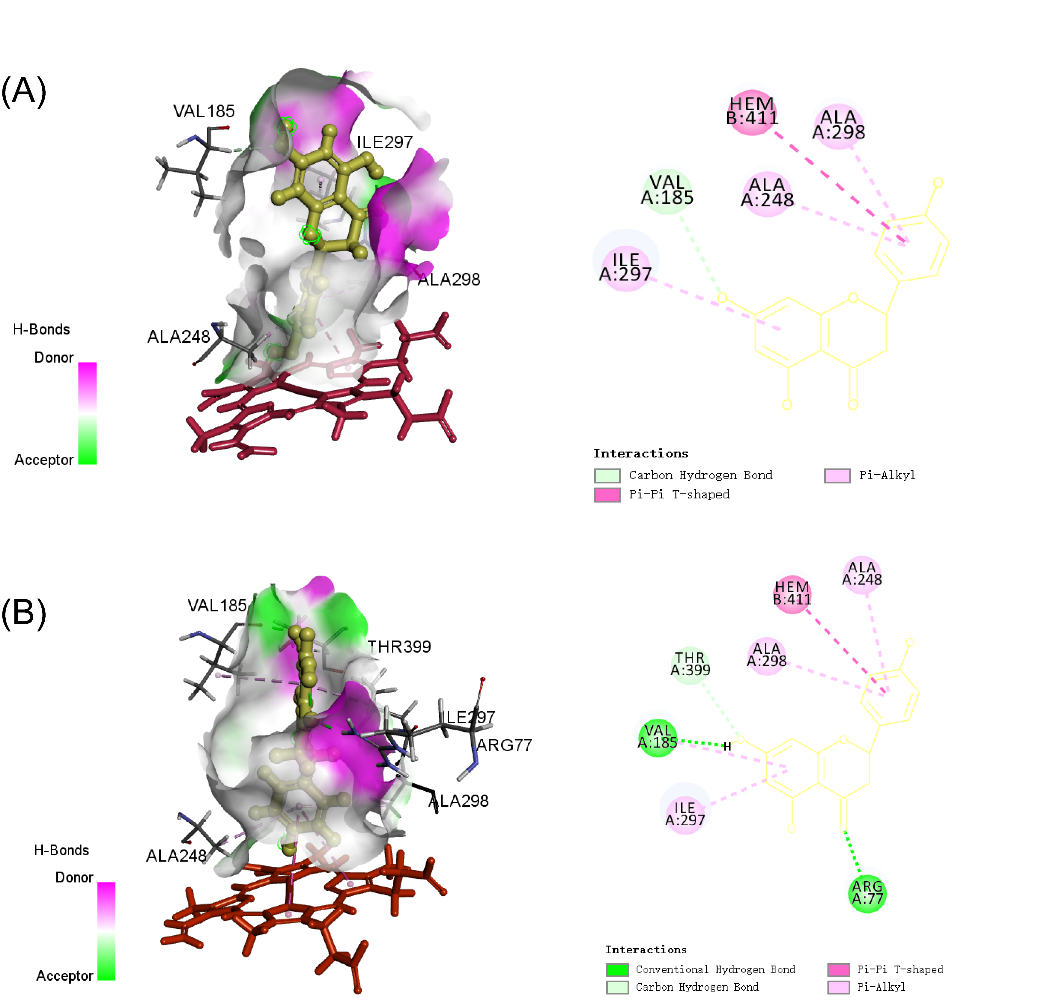
**

**Figure S3.** The interactions between naringenin and sac-2_mut_ model (**A**) and sca-2_mut_R88A/S96A model (**B**).

**
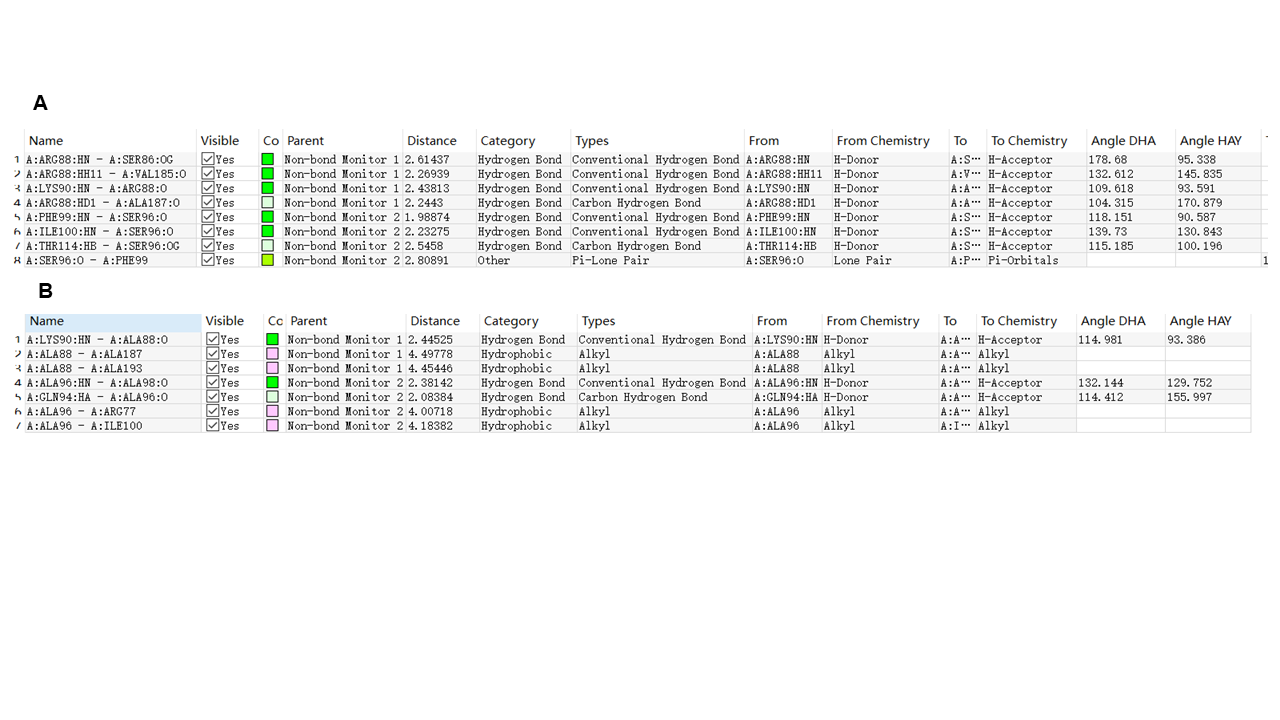
**

**Figure S4.** The interactions of position 88 and 96 amino acid with surrounding amino acids. (**A**) the predicted sca-2_mut_ model. (**B**) the predicted sca-2_mut_R88A/S96A model

**
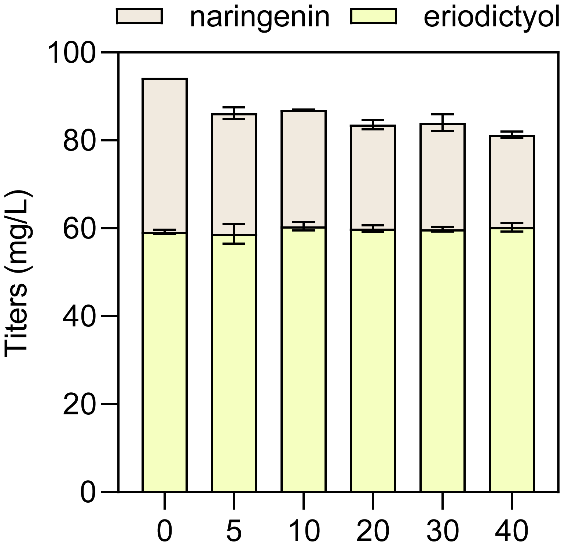
**

**Figure S5.** Effect of hemin addition on the titer of eriodictyol using the HFLA-19 strain. The data are shown as mean ± SD of three biological replicates.

**Supplementary Tables**

**Table S1. Plasmids used in the study.** Abbreviations: Km, kanamycin; Cm, chloramphenicol; R, resistance.

| Plasmids | Description | Source |
| --- | --- | --- |
| pRSFDuet-1 | Expression vector, RSF1030 *ori*, double T7 *lac* promoters; Km^R^ | Novagen |
| pACYCDuet-1 | Expression vector, p15A *ori*, double T7 *lac* promoters; Cm^R^ | Novagen |
| pRSF-BM3 | pRSFDuet-1derivative, *Bacillus Megatherium* BM3; Km^R^ | Lab stock |
| pRSF-105P2-CamA-CamB | pRSFDuet-1derivative, *Streptomyces peucetius* CYP105P2, *Pseudomonas putida camA* and *camB*; Km^R^ | This study |
| pRSF-105D7-CamA-CamB | pRSFDuet-1derivative, *Streptomyces avermitilis* CYP105D7, *Pseudomonas putida camA* and *camB*; Km^R^ | This study |
| pRSF-moxA_mut_- CamA-CamB | pRSFDuet-1derivative, *Nonomuraea recticatena moxA_mut_*, *Pseudomonas putida camA* and *camB*; Km^R^ | This study |
| pRSF-105A1_mut_- CamA-CamB | pRSFDuet-1derivative, *Streptomyces griseolus 105A1_mut_*, *Pseudomonas putida camA* and *camB*; Km^R^ | This study |
| pRSF-sca-2_mut_- CamA-CamB | pRSFDuet-1derivative, *Streptomyces carbophilus sca-2_mut_*, *Pseudomonas putida camA* and *camB*; Km^R^ | This study |
| pRSF-sca-2_mut_ | pRSFDuet-1derivative, *Streptomyces carbophilus sca-2_mut_*; Km^R^ | This study |
| pRSF-sca-2_mut_-Fdx_1499-FdR_0978 | pRSFDuet-1derivative, *S. carbophilus sca-2_mut_*, *Synechococcus elongates PCC7942 Fdx_1499* and *FdR_0978*; Km^R^ | This study |
| pRSF-sca-2_mut_-Fld-Fpr | pRSFDuet-1derivative, *S. carbophilus sca-2_mut_*, *E. coli Fld* and *Fpr*; Km^R^ | This study |
| pRSF-sca-2_mut_-FldA-Fpr | pRSFDuet-1derivative, *S. carbophilus sca-2_mut_*, *E. coli FldA* and *Fpr*; Km^R^ | This study |
| pRSF-sca-2_mut_-FldB-Fpr | pRSFDuet-1derivative, *S. carbophilus sca-2_mut_*, *E. coli FldB* and *Fpr*; Km^R^ | This study |
| pRSF-sca-2_mut_-YkuN-Fpr | pRSFDuet-1derivative, *S. carbophilus sca-2_mut_*, *Bacillus subtilis YkuN* and *E. coli Fpr*; Km^R^ | This study |
| pRSF-sca-2_mut_- YkuP-Fpr | pRSFDuet-1derivative, *S. carbophilus sca-2_mut_*, *B. subtilis YkuP* and *E. coli Fpr*; Km^R^ | This study |
| pRSF-sca-2_mut_-BM3 | pRSFDuet-1derivative, *S. carbophilus sca-2_mut_* fused with reductase domain of P450 BM3 from *Bacillus Megatherium*; Km^R^ | This study |
| sca-2_mut_ R77A-Fld-Fpr | pRSF-sca-2_mut_-Fld-Fpr derivative, sca-2_mut_ (R77A); Km^R^ | This study |
| sca-2_mut_ R88A-Fld-Fpr | pRSF-sca-2_mut_-Fld-Fpr derivative, sca-2_mut_ (R88A); Km^R^ | This study |
| sca-2_mut_ R93A-Fld-Fpr | pRSF-sca-2_mut_-Fld-Fpr derivative, sca-2_mut_ (R93A); Km^R^ | This study |
| sca-2_mut_ G95A-Fld-Fpr | pRSF-sca-2_mut_-Fld-Fpr derivative, sca-2_mut_ (G95A); Km^R^ | This study |
| sca-2_mut_ S96A-Fld-Fpr | pRSF-sca-2_mut_-Fld-Fpr derivative, sca-2_mut_ (S96A); Km^R^ | This study |
| sca-2_mut_ R197A-Fld-Fpr | pRSF-sca-2_mut_-Fld-Fpr derivative, sca-2_mut_ (R197A); Km^R^ | This study |
| sca-2_mut_ R88A/S96A-Fld-Fpr | pRSF-sca-2_mut_-Fld-Fpr derivative, sca-2_mut_ (R88A/S96A); Km^R^ | This study |
| ADB-N-sca-2_mut_ R88A/S96A-Fld-Fpr | pRSF-sca-2_mut_-Fld-Fpr derivative, ADB1-N-Fpr, ADB2-N-Fld, ADB3-N-sca-2_mut_ (R88A/S96A); Km^R^ | This study |
| ADB-C-sca-2_mut_ R88A/S96A-Fld-Fpr | pRSF-sca-2_mut_-Fld-Fpr derivative, ADB1-C-Fpr, ADB2-C-Fld, ADB3-C-sca-2_mut_ (R88A/S96A); Km^R^ | This study |
| DNA scaffold | pACYCDuet-1 derivative; scaffold1-scaffold2-scaffold3; Cm^R^ | This study |
| Lig-N-sca-2_mut_ R88A/S96A-Fld-Fpr | pRSF-sca-2_mut_-Fld-Fpr derivative, GBDlig-N-Fpr, SH3lig-N-Fld, PDZlig-N-sca-2_mut_ (R88A/S96A); Km^R^ | This study |
| Lig-C-sca-2_mut_ R88A/S96A-Fld-Fpr | pRSF-sca-2_mut_-Fld-Fpr derivative, GBDlig-C-Fpr, SH3lig-C-Fld, PDZlig-C-sca-2_mut_ (R88A/S96A); Km^R^ | This study |
| Protein scaffold | pACYCDuet-1 derivative; GBD-SH3-PDZ; Cm^R^ | This study |
| CipA-N-sca-2_mut_ R88A/S96A-Fld-Fpr | pRSF-sca-2_mut_-Fld-Fpr derivative, CipA-N-Fpr, CipA-N-Fld, CipA-N-sca-2_mut_ (R88A/S96A); Km^R^ | This study |
| CipA-C-sca-2_mut_ R88A/S96A-Fld-Fpr | pRSF-sca-2_mut_-Fld-Fpr derivative, CipA-C-Fpr, CipA-C-Fld, CipA-C-sca-2_mut_ (R88A/S96A); Km^R^ | This study |
| CipB-N-sca-2_mut_ R88A/S96A-Fld-Fpr | pRSF-sca-2_mut_-Fld-Fpr derivative, CipB-N-Fpr, CipB-N-Fld, CipB-N-sca-2_mut_ (R88A/S96A); Km^R^ | This study |
| CipB-C-sca-2_mut_ R88A/S96A-Fld-Fpr | pRSF-sca-2_mut_-Fld-Fpr derivative, CipB-C-Fpr, CipB-C-Fld, CipB-C-sca-2_mut_ (R88A/S96A); Km^R^ | This study |

**Table S2. The primers used in this study**

| Primers | Sequence (5'-3') ^a^ |
| --- | --- |
| Fdx_1499-RH-F | CTTTAATAAGGAGATATACCATGGCAACCTACAAGGTTACGCT |
| Fdx_1499-RH-R | CCTCCTTTTATGAGCTCGAATTCGGATCCTCAGTAGAGGTCTTCTTCTTTGTGGGT |
| Fdr_0978-RH-F | TGAGGATCCGAATTCGAGCTCATAAAAGGAGGAAAATATATGTTGAATGCGAGTGTGGCT |
| Fdr_0978-RH-R | GCGGCCGCAAGCTTGTCGACCTAGTAGGTTTCAACATGCCAACGACC |
| Fld-RH-F | TTTAATAAGGAGATATACCATGGCGGAAATTGGTATTTTTGTCGG |
| Fld-RH-R | CCTCCTTTTATGAGCTCGAATTCGGATCCTCAGGACAACAGCGTGCCCCA |
| FldA-RH-F | CTTTAATAAGGAGATATACCATGGCTATCACTGGCATCTTTTTCG |
| FldA-RH-R | CCTCCTTTTATGAGCTCGAATTCGGATCCTCAGGCATTGAGAATTTCGTCGAGATG |
| FldB-RH-F | CTTTAATAAGGAGATATACCATGAATATGGGTCTTTTTTACGGTTCCAGC |
| FldB-RH-R | CCTCCTTTTATGAGCTCGAATTCGGATCCTCAGGCGTAATGCTCTGCCATTT |
| YkuP-RH-F | CTTTAATAAGGAGATATACCATGGCGAAGATTTTGCTCGTTTATGC |
| YkuP-RH-R | CCTCCTTTTATGAGCTCGAATTCGGATCCTCAGACAGCGCACCCGCTT |
| YkuN-RH-F | CTTTAATAAGGAGATATACCATGGCTAAAGCCTTGATTACATATGCCA |
| YkuN-RH-R | CCTCCTTTTATGAGCTCGAATTCGGATCCTTATGAAACATGGATTTTTTCCTTGTTCATAT |
| Fpr-RH-F | TGAGGATCCGAATTCGAGCTCATAAAAGGAGGAAAATATATGGCTGATTGGGTAACAGGC |
| Fpr-RH-R | TGCGGCCGCAAGCTTGTCGACTTACCAGTAATGCTCCGCTGTCATATGG |
| Sca2-RH-F | TATAAGAAGGAGATATACATATGACCGAGATGACCGAAAAGGC |
| Sca2-RH-R | GCTCGGGCTCGGAATGCCACCCCAGGTAACCGGCAGTTCGT |
| BM3-RH-F | GGTGGCATTCCGAGCCCGAG |
| BM3-RH-R | GTTTCTTTACCAGACTCGAGTTAGTGGTGGTGGTGGTGGTGG |
| R77A-F | GCAGCGACGCGCTGCACGCGGATTTCCCG |
| R77A-R | GCGTGCAGCGCGTCGCTGCTCAGACGCGG |
| R88A-F | TTAGCCCGGCGATCAAAGCGTTCCGTCAGGGT |
| R88A-R | GCTTTGATCGCCGGGCTAAACGCCGGGAAAT |
| R93A-F | AAGCGTTCGCGCAGGGTAGCCCGGCGTTTA |
| R93A-R | CTACCCTGCGCGAACGCTTTGATACGCGGGCTAA |
| G95A-F | TCCGTCAGGCGAGCCCGGCGTTTATTGGCAT |
| G95A-R | GCCGGGCTCGCCTGACGGAACGCTTTGATACGC |
| S96A-F | GTCAGGGTGCGCCGGCGTTTATTGGCATGGACC |
| S96A-R | AACGCCGGCGCACCCTGACGGAACGCTTTG |
| R197A-F | CGGCGGCGGACGATTTTGAACGTTATCTGGACGG |
| R197A-R | TCAAAATCGTCCGCCGCCGCGTTCGCGCTAT |
| N-Fld-ADB2-F1 | CCATCGTCGTAGCAATCGCTGCAGCAATCGTGGTGGTGGTGGTTCTGCGGAAATTGGTATTTTTGTCGGCAC |
| N-Fld-ADB2-F2 | TTTAATAAGGAGATATACCATGGTGAGCAGCCGTCGTAGCCATCGTCGTAGCAATCGCT |
| N-Fld-ADB2-R | CCTCCTTTTATGAGCTCGAATTCGGATCCTCAGGACAACAGCGTGCCCCA |
| N-Fpr-ADB1-F1 | ATGCGTAGCAATCGTCGTGATCATACCGTGAGCACCCGTCAGAGCAATATTGG |
| N-Fpr-ADB1-F2 | TCCTGAGGATCCGAATTCGAGCTCATAAAAGGAGGAAAATATATGCGTAGCAATCGTCGTGATCAT |
| N-Fpr-ADB1-R | TGCGGCCGCAAGCTTGTCGACTTACCAGTAATGCTCCGCTGTCATATGG |
| N-Sca-ADB3-F1 | TAGCCACCGTCAGACCCATCAGGGTGGTGGTGGTTCTACCGAGATGACCGAAAAGGCGA |
| N-Sca-ADB3-F2 | GTATAAGAAGGAGATATACATATGCAGAGCAGCCGTCGTAGCCATCGTCGTAGCCACCGTCAGACCCATCAG |
| N-Sca-ADB3-R | AGCGGTTTCTTTACCAGACTCGAGTTAG |
| C-Fld-ADB2-F | TTTAATAAGGAGATATACCATGGCGGAAATTGGTATTTTTGTCGG |
| C-Fld-ADB2-R1 | TTGCTACGACGATGGCTACGACGGCTGCTCACAGAACCACCACCACCGGACAACAGCGTGCCCCAAT |
| C-Fld-ADB2-R2 | TGAGCTCGAATTCGGATCCTCAACGATTGCTGCAGCGATTGCTACGACGATGGCTACGACGGCT |
| C-Fpr-ADB1-F | GCAATCGTTGAGGATCCGAATTCGAGCTCATAAAAGGA |
| C-Fpr-ADB1-R1 | GCTCACGGTATGATCACGACGATTGCTACGAGAACCACCACCACCCCAGTAATGCTCCGCTGTCATATGG |
| C-Fpr-ADB1-R2 | GCGGCCGCAAGCTTGTCGACTTAAATATTGCTCTGACGGGTGCTCACGGTATGATCACGACGATT |
| C-Sca-ADB3-F | ATAAGAAGGAGATATACATATGACCGAGATGACCGAAAAGGCGA |
| C-Sca-ADB3-R1 | ACGATGGCTACGACGGCTGCTCTGAGAACCACCACCACCGTGGTGGTGGTGGTGGTGCCA |
| C-Sca-ADB3-R2 | GTTTCTTTACCAGACTCGAGTTACTGATGGGTCTGACGGTGGCTACGACGATGGCTACGACGGCTG |
| DNA-scaffold-F | CATAAGACGAGGGGGTGTATTCACGGAAGGGGGGGTACACTCGAGTCTGGTAAAGAAACCGCT |
| DNA-scaffold-R | AATACACCCCCTCGTCTTATGATTCTCCCTAGCTTGTAAGATCTGCCATATGTATATCTCCTTCTTATACT |
| N-Fld-SH3-F1 | GGCATTACCGCCGAAACGTCGTCGCGGTGGTGGTGGTTCTGCGGAAATTGGTATTTTTGTCGGCAC |
| N-Fld-SH3-F2 | CTTTAATAAGGAGATATACCATGCCGCCGCCGGCATTACCGCCGAAACGTCGT |
| N-Fld-SH3-R | CCTCCTTTTATGAGCTCGAATTCGGATCCTCAGGACAACAGCGTGCCCCA |
| N-Fpr-GBD-F1 | TTCATTCAAGCGATGAAGGCGAAGATCAGGCAGGTGATGAAGATGAAGATGGTGGTGGTGGTTCTGCTGATTGGGTAACAGGCAAAGT |
| N-Fpr-GBD-F2 | GGTGCCTTAATGCATGTTATGCAGAAACGCTCTCGCGCCATTCATTCAAGCGATGAAGGCGAAGATCAGGCAGGTGA |
| N-Fpr-GBD-F3 | TCCTGAGGATCCGAATTCGAGCTCATAAAAGGAGGAAAATATATGTTAGTGGGTGCCTTAATGCATGTTATGCAGAAAC |
| N-Fpr-GBD-R | TGCGGCCGCAAGCTTGTCGACTTACCAGTAATGCTCCGCTGTCATATGG |
| N-Sca-PDZ-F | ATAAGAAGGAGATATACATATGGGTGTGAAAGAATCTCTGGTTGGTGGTGGTGGTTCTACCGAGATGACCGAAAAGGCGA |
| N-Sca-PDZ-R | GTTTCTTTACCAGACTCGAGTTAGTGGTGGTGGTGGTGGTGCCAGG |
| C-Fld-SH3-F | TTTAATAAGGAGATATACCATGGCGGAAATTGGTATTTTTGTCGG |
| C-Fld-SH3-R | GCGACGACGTTTCGGCGGTAATGCCGGCGGCGGAGAACCACCACCACCGGACAACAGCGTGCCCCAAT |
| C-Fpr-GBD-F | ATTACCGCCGAAACGTCGTCGCTGAGGATCCGAATTCGAGCTCATAAAAGGAG |
| C-Fpr-GBD-R1 | AATGGCGCGAGAGCGTTTCTGCATAACATGCATTAAGGCACCCACTAAAGAACCACCACCACCCCAGTAATGCTCCGCTGTCATATGG |
| C-Fpr-GBD-R2 | GCGGCCGCAAGCTTGTCGACTTAATCTTCATCTTCATCACCTGCCTGATCTTCGCCTTCATCGCTTGAATGAATGGCGCGAGAGCGTTT |
| C-Sca-PDZ-F | TATAAGAAGGAGATATACATATGACCGAGATGACCGAAAAGGCGA |
| C-Sca-PDZ-R | GTTTCTTTACCAGACTCGAGTTAAACCAGAGATTCTTTCACACCAGAACCACCACCACCGTGGTGGTGGTGGTGGTGCCA |

^a^ Amino acid mutation sites are shown in red.

**Table S3. Strains applied and constructed in this study**

| Strains | Description | Source |
| --- | --- | --- |
| DH5α | *E. coli* str. K-12 F^–^ *endA1* *glnV44 thi-1 recA1 relA1 gyrA96 deoR nupG purB20* φ80d*lacZ*ΔM15 Δ(*lacZYA*-*argF*) U169 *hsdR17*(*r_K_*^–^*m_K_*^+^) λ^–^ | Invitrogen |
| BL21(DE3) | *E. coli str*. B F^–^ *ompT gal dcm lon hsdS_B_*(_rB_^–^_mB_^–^) λ (DE3 [*lacI lacUV5-T7p07 ind1 sam7 nin5*]) [*malB*^+^] _K-12_(λ^S^) | Novagen |
| C41(DE3) | Derived from BL21(DE3) by selecting for resistance to OGCP overexpression | Novagen |
| HFLA-1 | C41(DE3) harboring plasmid pRSF-105P2-CamA-CamB; Km^R^ | This study |
| HFLA-2 | C41(DE3) harboring plasmid pRSF-105D7-CamA-CamB; Km^R^ | This study |
| HFLA-3 | C41(DE3) harboring plasmid pRSF-moxA_mut_-CamA-CamB; Km^R^ | This study |
| HFLA-4 | C41(DE3) harboring plasmid pRSF-105A1_mut_-CamA-CamB; Km^R^ | This study |
| HFLA-5 | C41(DE3) harboring plasmid pRSF-sca-2_mut_-CamA-CamB; Km^R^ | This study |
| HFLA-6 | C41(DE3) harboring plasmid pRSF-sca-2_mut_-Fdx_1499-FdR_0978; Km^R^ | This study |
| HFLA-7 | C41(DE3) harboring plasmid pRSF-sca-2_mut_-Fld-Fpr; Km^R^ | This study |
| HFLA-8 | C41(DE3) harboring plasmid pRSF-sca-2_mut_-FldA-Fpr; Km^R^ | This study |
| HFLA-9 | C41(DE3) harboring plasmid pRSF-sca-2_mut_-FldB-Fpr; Km^R^ | This study |
| HFLA-10 | C41(DE3) harboring plasmid pRSF-sca-2_mut_-FkuN-Fpr; Km^R^ | This study |
| HFLA-11 | C41(DE3) harboring plasmid pRSF-sca-2_mut_-FkuP-Fpr; Km^R^ | This study |
| HFLA-12 | C41(DE3) harboring plasmid pRSF-sca-2_mut_-BM3; Km^R^ | This study |
| HFLA-13 | C41(DE3) harboring plasmid sca-2_mut_ R77A-Fld-Fpr; Km^R^ | This study |
| HFLA-14 | C41(DE3) harboring plasmid sca-2_mut_ R88A-Fld-Fpr; Km^R^ | This study |
| HFLA-15 | C41(DE3) harboring plasmid sca-2_mut_ R93A-Fld-Fpr; Km^R^ | This study |
| HFLA-16 | C41(DE3) harboring plasmid sca-2_mut_ G95A-Fld-Fpr; Km^R^ | This study |
| HFLA-17 | C41(DE3) harboring plasmid sca-2_mut_ S96A-Fld-Fpr; Km^R^ | This study |
| HFLA-18 | C41(DE3) harboring plasmid sca-2_mut_ R197A-Fld-Fpr; Km^R^ | This study |
| HFLA-19 | C41(DE3) harboring plasmid sca-2_mut_ R88A/S96A-Fld-Fpr; Km^R^ | This study |
| HFLA-20 | C41(DE3) harboring plasmids ADB-N-sca-2_mut_ R88A/S96A-Fld-Fpr and DNA scaffold; Km^R^, Cm^R^ | This study |
| HFLA-21 | C41(DE3) harboring plasmids ADB-C-sca-2_mut_ R88A/S96A-Fld-Fpr and DNA scaffold; Km^R^, Cm^R^ | This study |
| HFLA-22 | C41(DE3) harboring plasmids Lig-N-sca-2_mut_ R88A/S96A-Fld-Fpr and Protein scaffold; Km^R^, Cm^R^ | This study |
| HFLA-23 | C41(DE3) harboring plasmids Lig-C-sca-2_mut_ R88A/S96A-Fld-Fpr and Protein scaffold; Km^R^, Cm^R^ | This study |
| HFLA-24 | C41(DE3) harboring plasmids CipA-N-sca-2_mut_ R88A/S96A-Fld-Fpr; Km^R^ | This study |
| HFLA-25 | C41(DE3) harboring plasmids CipA-C-sca-2_mut_ R88A/S96A-Fld-Fpr; Km^R^ | This study |
| HFLA-26 | C41(DE3) harboring plasmids CipB-N-sca-2_mut_ R88A/S96A-Fld-Fpr; Km^R^ | This study |
| HFLA-27 | C41(DE3) harboring plasmids CipB-C-sca-2_mut_ R88A/S96A-Fld-Fpr; Km^R^ | This study |
